# State of the Art in Pulmonary Arterial Hypertension: Molecular Basis, Imaging Modalities, and Right Heart Failure Treatment

**DOI:** 10.3390/biomedicines13071773

**Published:** 2025-07-20

**Authors:** Melika Shafeghat, Yasmin Raza, Roberta Catania, Amir Ali Rahsepar, Blair Tilkens, Michael J. Cuttica, Benjamin H. Freed, Jingbo Dai, You-Yang Zhao, James C. Carr

**Affiliations:** 1 Radiology, Feinberg School of Medicine, Northwestern University, Chicago, IL 60611, USA; melika.shafeghat@northwestern.edu (M.S.); roberta.catania1@nm.org (R.C.); amirali.rahsepar@northwestern.edu (A.A.R.); 2 Cardiology, Feinberg School of Medicine, Northwestern University, Chicago, IL 60611, USA; yasmin.raza@northwestern.edu (Y.R.); blair.tilkens@nm.org (B.T.); benjamin.freed@northwestern.edu (B.H.F.); 3 Pulmonary and Clinical Care, Feinberg School of Medicine, Northwestern University, Chicago, IL 60611, USA; m-cuttica@northwestern.edu; 4 Program for Lung and Vascular Biology, and Section for Injury Repair and Regeneration Research, Stanley Manne Children’s Research Institute, Ann & Robert H. Lurie Children’s Hospital of Chicago, Chicago, IL 60611, USA; jingbo.dai@northwestern.edu (J.D.); youyang.zhao@northwestern.edu (Y.-Y.Z.); 5 Department of Pediatrics, Division of Critical Care, Northwestern University Feinberg School of Medicine, Chicago, IL 60611, USA

**Keywords:** pulmonary arterial hypertension, heart failure, BMPR2, magnetic resonance imaging

## Abstract

Pulmonary hypertension (PH) is broadly defined as a mean pulmonary arterial pressure (mPAP) exceeding 20 mm Hg at rest. Pulmonary arterial hypertension (PAH) is a specific subset of PH characterized by a normal pulmonary arterial wedge pressure (PAWP), combined with elevated mPAP and increased pulmonary vascular resistance (PVR), without other causes of pre-capillary hypertension such as lung diseases or chronic thromboembolic pulmonary hypertension. The majority of PAH cases are idiopathic; other common etiologies include connective tissue disease-associated PAH, congenital heart disease, and portopulmonary hypertension. To a lesser extent, genetic and familial forms of PAH can also occur. The pathophysiology of PAH involves the following four primary pathways: nitric oxide, endothelin-1, prostacyclin, and activin/bone morphogenetic protein (BMP). Dysregulation of these pathways leads to a progressive vasculopathy marked by vasoconstriction, vascular proliferation, elevated right heart afterload, and ultimately right-sided heart failure. Diagnosing PAH is challenging and often occurs at advanced stages. The gold standard for diagnosis remains invasive right heart catheterization. Along with invasive hemodynamic measurements, several noninvasive imaging modalities such as echocardiography and ventilation-perfusion scanning are key adjunct techniques. Also, recent advancements in cardiac magnetic resonance (CMR) have opened a new era for PAH management. Additionally, CMR and echocardiography not only enable diagnosis but also aid in evaluating disease severity and monitoring treatment responses. Current PAH treatments focus on targeting molecular pathways, reducing inflammation, and inhibiting right-sided heart failure. Integrating imaging with basic science techniques is crucial for enhanced patient diagnosis, and precision medicine is emerging as a key strategy in PAH management. Additionally, the incorporation of artificial intelligence into both molecular and imaging approaches holds significant potential. There is a growing need to integrate new imaging modalities with high resolution and reduced radiation exposure into clinical practice. In this review, we discuss the molecular pathways involved in PAH, the imaging modalities utilized for diagnosis and monitoring, and current targeted therapies. Advances in molecular understanding and imaging technologies, coupled with precision medicine, could hold promise in improving patient outcomes and revolutionizing the management of PAH patients.

## 1. Introduction

### 1.1. Overview of Pulmonary Arterial Hypertension

Pulmonary arterial hypertension (PAH) is Group I pulmonary hypertension (PH), a rare disease of the lung vasculature with a high (though improving) mortality rate [1]. The World Health Organization (WHO) categorizes PH into five distinct groups based on etiology, hemodynamic profile, and underlying mechanisms, with the common finding being a mean pulmonary arterial pressure (mPAP) over 20 mmHg. According to the updated 2022 guidelines from the European Society of Cardiology (ESC) and the European Respiratory Society (ERS), these groups are defined as follows: WHO Group I—PAH; WHO Group II—PH associated with left heart disease; WHO Group III—PH related to lung diseases and/or hypoxia; WHO Group IV—PH caused by chronic pulmonary artery obstructions/thrombosis, and WHO Group V—PH with unclear and/or multifactorial mechanisms [2].

Different clinical etiologies cause PAH (Table 1), including idiopathic (IPAH), heritable (due to gene mutations), or forms associated with other chronic diseases [2,3]. As a rare disease, it is hard to estimate the incidence and prevalence of all PAH cases regardless of subtypes. However, according to the latest Global Burden of Disease study, the estimated prevalence in 2021 was 192,000 patients worldwide, of which 62% were women [4].

Classically, the clinical presentation of PAH patients was mostly young women of childbearing age. In the current era, however, the average age of diagnosis of PAH has increased, possibly due to expanded awareness among clinicians and improvement in noninvasive screening [3,4].

### 1.2. Importance of Comprehending the Molecular and Hemodynamic Bases

It is important to underscore the molecular basis of PAH since the main pathology occurs at the endothelial cell level [5]. Plexiform lesions, or plexogenic arteriopathy, are vascular lesions in the medium to small muscular pulmonary arteries characterized by endothelial cell injury, proliferation, and apoptosis. While these lesions are more pathognomonic of PAH compared to other types of PH, remodeling with intimal and medial hypertrophy and development of fibrosis can occur across the entire PH clinical spectrum [6].

PAH is a form of pre-capillary PH that predominantly affects small arterioles. This distal arteriolar remodeling leads to increased right ventricular (RV) afterload, partly reflected by an increased pulmonary vascular resistance (PVR). Right heart catheterization (RHC) findings in PAH include an elevation in PVR to greater than 2 Wood units [7], along with an mPAP > 20 mm Hg and a normal pulmonary artery wedge pressure (PAWP) less than 15 mmHg [8].

### 1.3. Significance of Right-Sided Heart Failure in PAH

A marked increase in right heart afterload in patients with PAH, along with inflammation and neurohormonal activation, results in RV remodeling. Initial RV adaptation includes increases in wall thickness and contractility to maintain cardiac output (CO). Ultimately, these compensatory mechanisms can prove inadequate, with patients going on to develop a decrease in ventriculoarterial (VA) coupling and maladaptive remodeling, typified by eccentric hypertrophy, systolic dysfunction, and worsening tricuspid regurgitation [9]. Along with invasive hemodynamic assessment with RHC, imaging modalities remain highly relevant tools for diagnosing RV dysfunction. Indeed, echocardiography and cardiac magnetic resonance (CMR) have shown promising roles in diagnosing RV dysfunction and in complementing the assessment of clinical parameters, exercise capacity, and biochemical markers in determining overall PAH severity [10].

In this review, we aim to highlight the function of molecular pathways and imaging modalities in the diagnosis and treatment of patients with PAH. This narrative review is based on a literature search using PubMed, Google Scholar, and ClinicalTrials.gov, focusing on key English-language studies from the past 5–10 years on PAH, its mechanisms, current treatments, and CMR imaging. Articles were selected based on clinical relevance and author expertise.

## 2. Molecular Basis in PAH

### 2.1. Genetic Factors and Mutation

PAH has a high fatality rate, necessitating early diagnosis to prevent mortality. Moreover, 6 to 10% of PAH patients who are not considered PAH-related to other disorders have a positive family history of the disease. In 2000, the first genetic study revealed that such patients have heterozygous germline mutations in the bone morphogenetic protein receptor type 2 (BMPR2) gene, a member of the transforming growth factor-β (TGF-β) family [11,12]. Additional studies recognized the same mutation in IPAH patients as well. To date, BMPR2 mutations are accountable for 70 to 80% of heritable PAH (HPAH) cases and are noted in 10 to 20% of IPAH patients. Over 20 gene mutations were detected in IPAH or HPAH patients [13], with 12 of definitive evidence, 3 with moderate evidence, and 6 with limited evidence [14].

Notably, younger women are more affected, experience greater symptom severity at the time of diagnosis, and are more likely to be candidates for being screen positive for a mutation. Such evidence underscores the role of genomics in PAH patients.

### 2.2. Pathophysiology at the Molecular Level

PAH is characterized by histopathological changes primarily occurring at the level of small pulmonary arterioles. These changes include intimal damage and proliferation due to oxidative stress, and arteriolar medial hypertrophy. Fibrinoid necrosis and microthrombi are other common findings [15].

The definitive characteristic of PAH is the presence of plexiform lesions, described as complex structures composed of vascular channels formed by neoplastic endothelial cell proliferation [15,16]. The cellular composition of the plexiform lesions comprises at least two populations of phenotypically distinct endothelial cells, with a quiescent endothelial layer lining the channels and a de-differentiated, proliferating, apoptosis-resistant, and endothelial-to-mesenchymal transitioning (Endo-MT) phenotype at the core of the lesion [17].

Furthermore, specific cytokine levels, interleukin-1β and interleukin-6 (IL-6), are elevated in the serum of PAH patients, indicating the role of inflammation. The presence of inflammatory cells at the adventitia is another key indicator of PAH. Macrophages are the dominant inflammatory cells in both the RV and pulmonary vasculature, and they can be polarized to either M1 (proinflammatory) or M2 (anti-inflammatory) [18]. However, our unpublished data showed that macrophage polarization per se is unlikely to be related to pulmonary vascular remodeling.

### 2.3. Key Molecular Pathways

Current evidence from both mouse and human models has shown significantly higher numbers of pericytes—regulatory cells around the vessel walls—in the distal pulmonary vasculature, due to abnormal signaling pathways involving fibroblast growth factor 2 (FGF2) and IL-6 in dysfunctional endothelial cells. These pericytes can differentiate into smooth muscle-like cells, contributing to the pathology of PAH [19]. Additionally, mutations in BMPR2, a member of the TGF-β superfamily, further contribute to the uncontrolled proliferation of smooth muscle cells and fibroblasts, leading to fibrosis and scarring [20].

The TGF-β/BMP signaling pathway is one of the most studied and promising targets for developing new therapies for PAH [21]. Three major receptors related to the TGF-β/BMP pathway involved in PAH are activin receptors, TGF-β receptors, and BMPR2. These receptors, along with their ligands, regulate SMAD proteins, which subsequently drive transcriptional activity. SMAD2 and SMAD3, activated by TGF-β and activin signaling, promote smooth muscle cell proliferation and vascular remodeling. In contrast, SMAD1, SMAD5, and SMAD8, which are activated through BMPR2 signaling, exert protective effects by inducing apoptosis and maintaining vascular homeostasis. However, in the presence of BMPR2 mutations, SMAD1/5/8 signaling is significantly reduced, disrupting this balance and exacerbating the pathological processes underlying PAH [22].

**Figure 1:**
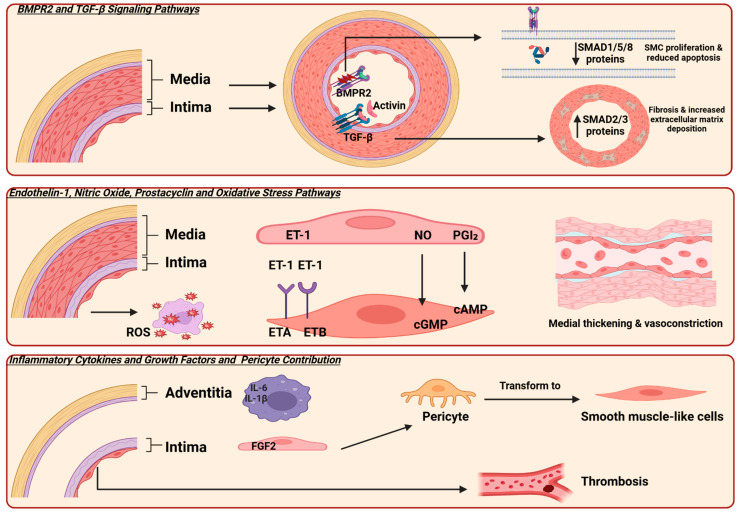
Illustrates the molecular pathways involved in the pathogenesis of pulmonary arterial hypertension (PAH). Bone morphogenetic protein receptor type 2 (BMPR2) and transforming growth factor-β (TGF-β) are the primary signaling pathways. BMPR2 is expressed on endothelial cells, and mutations in this receptor lead to decreased SMAD 1,5, and 8 signaling, resulting in smooth muscle cell proliferation and reduced apoptosis. Additionally, TGF-β is expressed in the smooth muscle cells of vessels, causing an increase in SMAD 2 and 3 protein expression, which leads to fibrosis and stiffness. Three key pathways involved in vascular tone regulation are endothelin-1 (ET-1), nitric oxide (NO), and prostacyclin (PGI_2_). In PAH, elevated ET-1 levels and dysregulation of its receptors contribute to vasoconstriction. Activation of the ETA receptor mediates vasoconstriction and smooth muscle proliferation, while the ETB receptor promotes vasodilation. An imbalance between these receptor activities favors vasoconstriction in PAH [25]. Additionally, mutations or dysfunction in the NO–cGMP pathway and reduced production of prostacyclin impair vasodilation, further contributing to disease progression. Furthermore, the presence of inflammatory cells in the adventitia and fibroblast growth factor 2 (FGF2) promotes the transformation of precursor cells into smooth muscle cells. Additionally, microthrombosis in small vessels is another characteristic finding. SMAD originates from the similarity to two gene families: the SMA genes (linked to the “small” phenotype in *Caenorhabditis elegans*) and the MAD genes (“Mothers Against Decapentaplegic”) found in *Drosophila*; reactive oxygen species (ROS); cyclic adenosine monophosphate (cAMP); cyclic guanosine monophosphate (cGMP); interleukin-6 (IL-6); interleukin-1β (IL-1β). Created in Biorender. https://app.biorender.com/ (accessed on 10 July 2025).

Furthermore, an imbalance in vasoactive mediators is a key factor contributing to the progression of PAH [23]. Three key vasoactive mediator pathways involved in the pathogenesis of PAH are the endothelin-1 (ET-1), nitric oxide (NO), and prostacyclin (PGI_2_). Dysregulation of these pathways leads to vasoconstriction and smooth muscle cell proliferation [24] (Figure 1).

Hypoxia is another key factor in the development of PAH by synergizing with other molecular pathways to accelerate vascular remodeling and disease advancement [26]. The hypoxia-inducible factor (HIF) pathway, specifically HIF-1α [27] and HIF-2α [28], regulates many pathogenic downstream factors or pathways, including vasomodulatory factors, inflammation, growth factors, and metabolic and mitochondrial dysregulation [26].

Although extensive research has been performed, the molecular mechanisms underlying the initiation and progression of PAH are still not fully elucidated. Recently, more novel pathways have been reported to participate in the development of PAH, including epigenetic regulators of gene transcription and translation [29,30], non-coding RNAs [31], RNA modifications [32], and ferroptosis [33].

## 3. Hemodynamic Basis of PAH

### 3.1. Hemodynamic Factors in PAH

Beyond the clinical presentation, the primary definition of PH is a resting mPAP greater than 20 mmHg, as determined by RHC. Additionally, both PAWP and PVR are utilized to differentiate across the various forms of PH, including pre-capillary, isolated post-capillary, and combined pre- and post-capillary.

According to the previous guidelines, pre-capillary PH used to be defined as PVR ≥ 3 Wood units, while isolated post-capillary was defined as PVR < 3 [34]. Along with the adjustment of mPAP threshold from 25 mmHg to 20 mmHg—based on upper normal range values in healthy individuals—the ESC and the ERS now consider a PVR above 2 Wood units as elevated in PH patients. Considering lower thresholds for mPAP and PVR emphasizes the importance of early diagnosis of PH [2]; however, current therapies for PAH have been investigated in populations using the previous thresholds.

### 3.2. Pulmonary Vascular Alterations

The main consequences of the above-mentioned insults on endothelial and smooth muscle cells are vasoconstriction, an increase in afterload, the formation of occlusive lesions, and an increase in wall shear stress.

Additionally, stiffening of the pulmonary artery (PA) has been acknowledged as the very first characteristic, pathophysiological mechanism, and predictor of severity in PH [35,36].

In both the distal pulmonary vasculature and the proximal PAs, vascular stiffening might occur as a result of pulmonary remodeling, which reduces their ability to dilate in response to blood flow within the vessels. Structural remodeling leads to decreased compliance in the larger pulmonary vasculature, resulting in an abnormal, chaotic blood flow pattern within the proximal PA. Reiter et al. have shown that vortical flow patterns occur in the MPA of patients with PAH more frequently than in healthy controls [37]. Also, increased PVR in distal vessels is another major concern. These are all consequences of mechanical and hemodynamic coupling [38].

### 3.3. Influence on Right-Sided Heart Function

Under normal physiological conditions, the pulmonary circulation functions at lower pressures compared to the systemic circulation; the anatomy of the thin-walled, crescent-shaped RV reflects this physiological context [39].

In pathological conditions such as PAH, increases in PVR and vascular remodeling lead to elevated RV wall stress. Initially, the RV undergoes hypertrophy as an adaptive response to maintain stroke volume by reducing wall stress, as described by the Law of LaPlace, which states that wall stress increases with chamber radius and decreases with wall thickness [40]; however, with maladaptive remodeling in severe cases, it eventually dilates, resulting in RV failure [41].

Furthermore, both main pulmonary artery (MPA) dilation and increased stiffness in PAH can augment the afterload of the pulmonary circulation and exacerbate RV stress. RV pressure-volume loop analysis is the key method to evaluate the coupling of RV-PA. RV-PA coupling refers to the interaction between afterload and ventricular contractility and is the marker of the RV’s adaptation to afterload. Conversely, RV-PA uncoupling signifies impaired RV function and an inadequate response to elevated pulmonary arterial afterload [42]. RV failure in PAH patients is a major contributor to morbidity.

## 4. Relation Between Molecular and Hemodynamic Factors

### 4.1. Interaction Between Molecular and Hemodynamic Mechanisms

Preclinical studies have shown that wall shear stress (WSS), the main mechanical force of blood affecting endothelial cells, is decreased in the MPA in PAH patients compared to controls. This reduction in WSS correlates with collagen deposition in the PA [43]. Additionally, a reduction in WSS contributes to decreased NO production from endothelial cells, which is another key characteristic of PAH and leads to vasoconstriction. A clinical study showed that decreased WSS, measured using velocity-encoded four-dimensional cardiac magnetic resonance imaging (4D-flow MRI) in PH patients, correlates with increases in hemodynamic parameters from right heart catheterization, such as PVR and mPAP [44].

Also, the presence of proinflammatory fibroblasts in the adventitia and imbalanced deposition of collagen to elastin in the extracellular volume (ECV) are other causes of PA stiffness, which can lead to decreased PA compliance with flowing blood and change the hemodynamics of the pulmonary circulation [36].

### 4.2. Implications for Disease Progression

In addition to the interaction of molecular and hemodynamic changes in the pulmonary vascular system, the RV undergoes critical remodeling as well. Several pathophysiological changes occur in the RV of patients with PAH.

RV hypertrophy, in response to high afterload, leads to an increase in the oxygen demand of hypertrophied myocytes, which further causes myocardial RV dysfunction. Notably, there is a key difference between RV and left ventricle (LV) failure; the RV under elevated wall stress is more vulnerable to oxidative stress, has diminished angiogenic compensation, and is more likely to initiate cell death mechanisms compared to the stressed LV [45].

Additionally, in animal experiments of HPAH with a positive BMPR2 mutation, triglyceride and ceramide deposition are seen in the RV and cause lipotoxicity due to the impaired fatty acid beta-oxidation. This evidence hallmarks that the lipotoxicity of the RV is associated with RV failure (RVF) in PAH patients [46].

Furthermore, another study showed that RV inflammation might even happen independently of RV dysfunction due to the increase in afterload. A preclinical study by Qazazi and colleagues showed that NLRP3 (nucleotide-binding domain, leucine-rich-containing family, pyrin domain-containing-3) macrophage activation in the RV leads to fibrosis and RVF in PAH. In the PAH model, macrophages exclusively accumulated in the RV, with no increase observed in the LV [47]. The current evidence highlights the importance of assessing the RV as a main contributor to mortality and the role of inflammation in the progression of the disease.

## 5. State-of-the-Art Imaging Approaches

### 5.1. Advanced Imaging Techniques

The gold standard method for diagnosing PH is pressure measurement through right heart catheterization, an invasive procedure. Consequently, several noninvasive imaging modalities have been introduced for the diagnosis and assessment of prognosis in PH patients. We aim to review the latest imaging methods, including echocardiography, ventilation-perfusion scans, computed tomography, and cardiac magnetic resonance (Table 2).

### 5.2. Echocardiography

Echocardiography, a noninvasive imaging technique, has increasingly been utilized for screening patients with PH. Regardless of the type of PH, echocardiography can provide various morphological and anatomical information, particularly the geometry of all cardiac chambers, including the right atrium, RV, and LV, as well as valvular structures and function. Common findings in PH patients include an enlarged RV visible in the parasternal long axis view and a dilated RV with an increased basal RV/LV ratio > in the apical 4-chamber view [2,48]. Interestingly, current evidence showed that the right atrial volume index to left atrial volume index correlated with mortality in PH patients (odds ratio: 1.91) [49].

**Figure 2:**
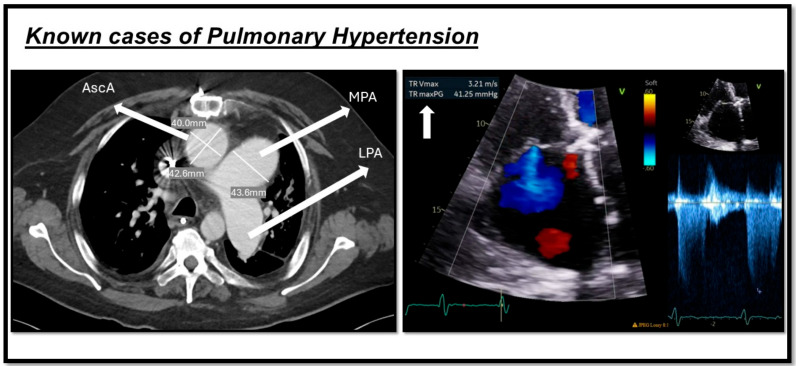
Shows two cases of pulmonary hypertension. The image on the left indicates a CT scan of one of our patients, demonstrating a dilated main pulmonary artery (MPA) measuring more than 29 mm, along with an increased MPA-to-aorta ratio (>1). The image on the right depicts echocardiography from another patient with pulmonary hypertension, showing an increase in tricuspid regurgitation velocity (TRV) as a marker of the condition. This figure is presented as part of an educational poster session at Radiology Society of North America (RSNA 2024). There is no copyright issue. 2024, Shafeghat. M.

Additionally, a key Doppler echocardiography measurement is tricuspid regurgitation velocity (TRV), with a TRV of >2.8 m/s indicating an at least intermediate risk of PH, and >3.4 m/s indicating a high risk [50]. Also, echocardiography can be used to estimate the hemodynamic parameters in a noninvasive approach. Systolic pulmonary arterial pressure (PASP) can be estimated by calculating the right ventricular systolic pressure (RVSP) using the TRV in the modified Bernoulli equation. In the absence of right ventricular outflow tract (RVOT) obstruction or pulmonary valve stenosis, PASP is considered equivalent to RVSP [51]. A current systematic review and meta-analysis showed that PASP has a sensitivity of 87% for a median cut-off of 36 mmHg for PH diagnosis [52]. However, according to the 2022 ESC/ERS guidelines on PH, peak TRV—not estimated PASP—should be used to assess the probability of PH [2] (Figure 2).

Echocardiography is a beneficial tool for assessing RV function measures, including tricuspid annular plane systolic excursion (TAPSE), RV fractional area change (RV-FAC), RV ejection fraction (RVEF), as well as strain measurement of RV in the lateral free wall (FWS) [53]. Additionally, the TAPSE/PASP ratio, derived from echocardiography, serves as a noninvasive measure of RV–PA coupling [54] and could be utilized in the diagnosis of PH [55].

Another useful finding is the pattern of RV outflow tract (RVOT) blood flow, where mid-systolic notching can represent the presence of pre-capillary PH, such as PAH [56]. A study by Arkles demonstrated that the presence of mid-systolic notching, compared to late systolic notching, has greater specificity for assessing elevated PVR [57]. Another study by Dong and colleagues indicated the role of echocardiography beyond just diagnosis and explored its prognostic role by examining the right atrial area, pericardial effusion, and TAPSE in the evaluation of PAH patients [58]. Echocardiography is the key imaging modality for baseline screening and follow-up assessment in patients with PAH [59].

It is worthwhile considering the absolute TRV value in combination with other echocardiographic signs of PH, including RV dilatation, RV dysfunction, and interventricular septal flattening, rather than relying solely on ePASP in clinical practice. Regardless of the noninvasive technique and the beneficial role of echocardiography in the initial assessment and prognosis of PH, it has several limitations. Echocardiography is operator-dependent for the identification of the maximal TRV. Obtaining adequate acoustic windows to visualize the entire RV can often be difficult. The abnormal shape of the RV impedes accurate quantitative assessment of the RV size and function [60]. Tricuspid regurgitation needs to be consistently present to measure TRV and, as a result, may overlook PH in patients without TR. There is still, therefore, a gap in the independent use of echocardiography as a surrogate method [61].

### 5.3. Computed Tomography

Computed tomography (CT) is an important diagnostic modality for the assessment of PH. It provides detailed imaging of both the heart and lungs, aiding in the diagnosis and understanding of underlying conditions that may contribute to PH.

Non-contrast CT (NCCT) is particularly useful for assessing lung parenchyma, and contrast CT is utilized for detecting the presence of chronic pulmonary embolism, which corresponds to WHO Groups III and IV PH. Additionally, CT is utilized to accurately diagnose a variety of thoracic diseases that can induce PH, such as fibrosing mediastinitis, sarcoidosis, and Langerhans cell histiocytosis. The main diagnostic parameter derived from CT for PH diagnosis is PA morphology: a dilated PA measuring greater than 29 mm and an increased PA-to-aortic ratio exceeding 1 [62]. There is not enough evidence to support the diagnostic accuracy of CT in PH patients. However, a study on chronic thromboembolic pulmonary hypertension (CTEPH) patients showed that CT-based PA morphology has a sensitivity of 0.98 and a specificity of 0.99 [63].

PA dilation is a common finding in patients with PH. Several studies have demonstrated a correlation between PA dilation and hemodynamic parameters, particularly mPAP. For example, a study by Duijnhouwer and colleagues reported a modest correlation between PA diameter and mPAP (r = 0.23, *p* < 0.001) [64].

Research also indicates that PA dilation may vary among different PH groups, including subgroups of PAH and CTEPH. A study by Żyłkowska found that the largest PA diameter was observed in patients with congenital heart defect-associated PAH (42.6 ± 7.6 mm) [65]. Additionally, PA dilation has been suggested as a potential independent predictor of mortality in PAH and CTEPH, likely due to its association with RV failure. Mechanisms for such increased risk include PA compression of the left main coronary artery, and PA dissection, or rupture caused by damage to the vessel wall [65].

A study by Xi and colleagues reinforces these findings, showing that patients with congenital heart disease-associated PAH exhibit the largest PA diameters and the highest PA-to-aortic ratios. CTEPH patients, while demonstrating the second-largest PA diameters, had lower PA/Ao ratios due to the relative enlargement of the aorta in this group [66].

However, there are limitations to using PA dilation as a marker. For instance, a standardized guideline is lacking for the specific site of PA measurement. Moreover, PA diameter is influenced by factors such as body surface area, sex, and age [67]. For example, normal PA diameters in healthy Chinese individuals have been reported as 22.41 ± 2.59 mm (range: 17.31–27.47 mm) [68]. Therefore, the PA-to-aortic ratio may be a more reliable parameter for assessing PA dilation, as it accounts for individual variations.

Additionally, there are other CT scan findings in PAH, including mosaic attenuation and ground-glass opacification (GGO). Mosaic attenuation is a broader term that refers to patchy areas of varying lung attenuation. In the context of pulmonary vascular diseases, primarily PH, it is most commonly seen in CTEPH and PAH cases [69]. The patchy attenuation in mosaic patterns typically corresponds to reduced blood flow in areas of decreased attenuation [70].

In contrast, GGO refers to an area of increased lung parenchymal attenuation that still allows visualization of the underlying bronchial and vascular structures. In the context of PAH, GGOs mostly occur as secondary changes, such as pulmonary vascular congestion, microvascular injury, or edema resulting from elevated PAP [69,71]. Additionally, the presence of GGO may be a consequence of pulmonary hemorrhage. In such cases, macrophages may endocytose the debris from red blood cells, leading to the formation of granulomas [72].

A study by Rajaram demonstrated that GGOs in the PAH group were most commonly observed in PAH associated with congenital heart disease (PAH-CHD) [73]. Regarding GGO patterns and distribution, most PAH patients exhibited a centrilobular pattern compared to a central pattern [73]. Another study indicated that centrilobular GGOs are correlated with worse outcomes in PAH patients [74].

In PAH, CT scan imaging can be utilized for both diagnosis and prognosis. Particularly, the presence of centrilobular GGO on a CT scan of IPAH patients showed negative prognostic value in terms of 2-year outcomes [75]. The main drawback regarding CT scans is the exposure to ionizing radiation, which limits their use in routine clinical practice and for follow-up of patients [76].

The newest modality is dual-energy CT (DECT), which uses advanced CT protocols to generate and reconstruct comprehensive maps of both perfusion and ventilation in the pulmonary circulation. Its primary advantage is that it consolidates imaging into a single modality, unlike V/Q SPECT, while providing high spatial resolution and more detailed anatomical and structural information. Additionally, it provides color-coded perfusion maps showing relative blood flow in different lung regions, aiding in the assessment of disease severity [77].

In a study by Giordano, CT perfusion was abnormal in 100% of CTEPH patients, while 52.6% of PAH patients had abnormal CT perfusion. Additionally, in most PAH patients, V/Q scans appeared normal or indicated a speckled pattern, yet did not show the characteristic perfusion defects of CTEPH patients [78]. This study indicated that CT perfusion findings are concordant with V/Q observations. So far, CT perfusion has shown excellent agreement with V/Q for the diagnosis of CTEPH [79]. Regarding PAH, the most common pattern observed in their CT perfusion was a patchy perfusion defect [80]. While DECT offers enhanced visualization of the lung parenchyma, it relies on the use of iodinated contrast agents.

### 5.4. Nuclear Medicine

Two main traditional nuclear medicine techniques used to evaluate pulmonary function are planar ventilation-perfusion (V/Q) scanning and V/Q SPECT (single photon emission computed tomography), with the latter using a rotating gamma detector camera to generate three-dimensional images [81]. Historically, V/Q scintigraphy has been the primary method for distinguishing CTEPH from other forms of PH, particularly PAH [82]. There are several V/Q patterns; defects in perfusion with normal ventilation may be related to CTEPH or other possible diagnoses, including fibrosing mediastinitis or tumor. In individuals without parenchymal lung disease, a normal perfusion scan effectively rules out CTEPH with a negative predictive value of 98% [83]. On the other hand, matched V/Q defects are observed in individuals with chronic lung diseases, classified as Group 3.

### 5.5. Cardiac Magnetic Resonance

The revolutionary use of cardiac magnetic resonance (CMR) is in its ability to obtain a wide range of information using a single modality. It provides anatomical and morphological details, functional assessment, as well as wall motion analysis, perfusion, viability, and angiography [84].

**Figure 3:**
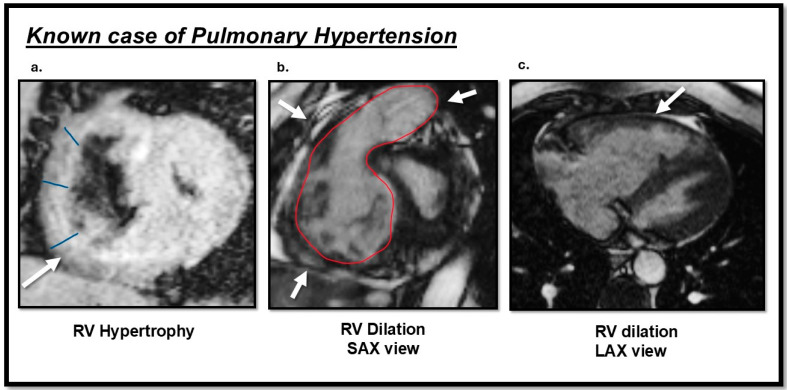
Cardiac magnetic resonance (CMR) TRUFI and cine images sequences findings of a patient with pulmonary hypertension (PH): (**a**) the arrow indicates right ventricular (RV) hypertrophy in the short-axis view; (**b**) RV dilation observed in the short-axis view; (**c**) RV dilation observed in the long-axis view. This image was obtained from routine clinical imaging. There is no copyright issue. Courtesy by O. McCarthy 2024.

CMR provides different information based on the designated imaging sequence and the specific purpose of the examination. The cine sequence is most frequently used to assess cardiac function. Several studies on PH patients evaluated RV function, especially the RVEF, as the RV attempts to adapt to the high PA afterload and is an important indicator of severity [85]. A recent meta-analysis on the predictive value of RV volumetric parameters in PAH patients revealed that a 1% reduction in RVEF is associated with a 2.1% increased risk of mortality over a 54-month period and a 4.9% increased risk of clinical worsening over a 22-month follow-up period [86]. Additional findings in PH patients include RV hypertrophy, an increased RV/LV ratio, RV dilation, septal flattening and bowing, functional tricuspid regurgitation, and right atrial dilation, all of which correlate with disease severity (Figure 3) [2].

**Figure 4:**
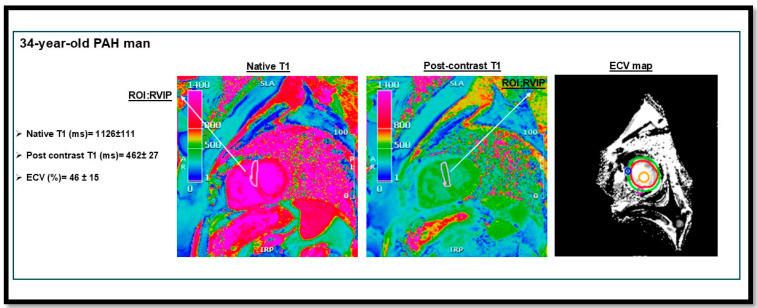
T1 mapping and extracellular volume (ECV) measurement in a patient with pulmonary arterial hypertension (PAH) from our institutional cohort. The image demonstrates elevated native T1 and ECV values at the right ventricular insertion points (RVIPs). RVIP T1 values (ms) in PAH compared to controls: 1084 ms [95% CI, 1071–1097] vs. 999 ms [95% CI, 985–1014] [89]. This figure is presented as part of an educational poster session at Radiology Society of North America (RSNA 2024). There is no copyright issue. 2024, Shafeghat. M.

Regarding tissue characterization techniques, T1 mapping, T2 mapping, and late gadolinium enhancement (LGE) are mainly used in PH patients. T1 mapping generates pixel-by-pixel data representing longitudinal relaxation times (T1 time), which vary based on tissue properties. Regarding myocardium, T1 values might be higher than normal in the context of high collagen deposition in the interstitial layers or, ultimately, in fibrosis and scars [87]. T1 mapping and ECV measurements are mostly performed in the IPAH population. PAH and CTEPH patients are at higher risk of RV fibrosis and failure compared to other PH etiologies, as the RV is subjected to significantly increased afterload [88]. A recent study showed that T1 values are 9% higher in PAH patients than in controls in the RV insertion points (RVIPs), with both T1 and ECV showing the greatest increases (Figure 4) [89]. LGE imaging is another well-established technique for tissue characterization; gadolinium accumulates in interstitial tissue and washes out without entering healthy cells [90]. In PH, two common types of LGE are observed, as follows: enhancement of the interventricular septum and the RVIPs [91]. Freed and colleagues demonstrated that LGE enhancement in RVIP is correlated with markers of disease severity, including increased RV volume, decreased RVEF, and higher mPAP in a cohort of 58 PH patients [92].

**Figure 5:**
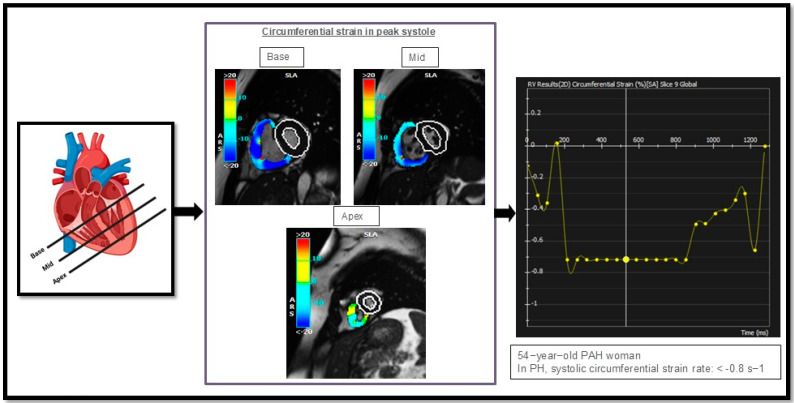
Illustrates circumferential strain rate measurements in the right ventricle (RV) of one of our PAH cases, analyzed in three different regions: the basal, mid, and apical segments of the heart. This figure is presented as part of an educational poster session at Radiology Society of North America (RSNA 2024). There is no copyright issue. 2024, Shafeghat M.

Another component of CMR is strain measurement. Current studies have shown that reductions in RV strain measurements—in circumferential, longitudinal, and radial directions—are hallmarks of impaired contractility, signifying RV dysfunction in PAH as the RV struggles to effectively pump blood against increased afterload (Figure 5) [93].

**Figure 6:**
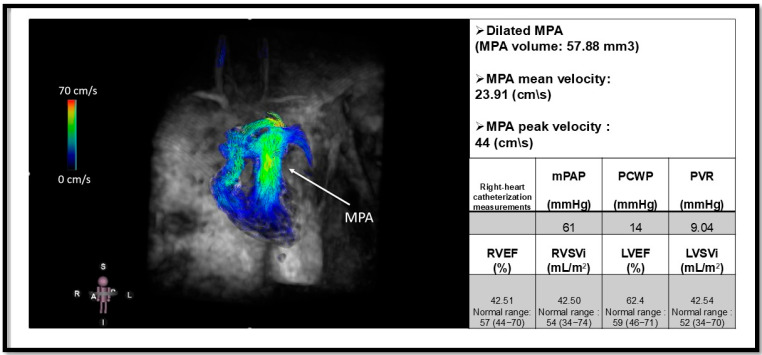
Four-dimensional flow MRI (4D flow MRI) of a patient with pulmonary arterial hypertension (PAH) and an elevated mean pulmonary arterial pressure (mPAP) of 61 mmHg. The arrow indicates a dilated main pulmonary artery (MPA). This image illustrates the utility of 4D flow MRI in visualizing flow patterns within the MPA and its branches, including the right pulmonary artery (RPA) and left pulmonary artery (LPA). Additionally, quantitative measurements such as peak and mean velocities can be obtained using 4D flow MRI [95]. The mean velocity of MPA is reduced in our patient. Functional metrics are derived from Cine cardiac magnetic resonance. Pulmonary capillary wedge pressure (PCWP); pulmonary vascular resistance (PVR); right ventricular ejection fraction (RVEF); Right ventricular stroke volume index (RVSVi); Left ventricular ejection fraction (LVEF); left ventricular stroke volume index (LVSVi). This figure is presented as part of an educational poster session at Radiology Society of North America (RSNA 2024). There is no copyright issue. 2024, Shafeghat M.

The newest MR imaging technique, three-directional velocity-encoding time-resolved MRI, or 4D flow MRI, measures velocity in three spatial vectors over time. After acquiring data within a three-dimensional volume, physicians have the flexibility to analyze and quantify blood flow dynamics in any region of interest [94]. This enhances versatility and allows for detailed assessment of hemodynamics, providing a more comprehensive understanding of blood flow patterns and velocities. The main advantages of 4D flow MRI include flow visualization and the provision of anatomical and hemodynamic information (Figure 6).

A recent study by Crene and colleagues showed that peak and mean velocities were significantly lower in PAH patients compared to controls at the left pulmonary artery (LPA), with values of 36 ± 12 cm/second and 20  ±  4 cm/second versus 59  ±  15 cm/second and 32  ±  9 cm/second, respectively. They also observed significant correlations between peak velocities and RVEF at all three vessels, that is, the main pulmonary artery (MPA) (r = 0.286), right pulmonary artery (RPA) (r = 0.400), and LPA (r = 0.401) [95]. Interestingly, Reiter et al. demonstrated that, beyond estimating mPAP from the duration of vortical flow using a regression model [37], the duration of the vortex within the MPA can also be used to track disease progression by comparison to baseline RHC measurements. They additionally validated their model using the newer diagnostic threshold of mPAP > 20 mmHg [96]. The recent study by Elbaz et al. demonstrated that quantitative 4D flow MRI-derived metrics, including kinetic energy and energy loss within LPA, can effectively differentiate PAH from Group II PH secondary to left heart disease. Notably, PAH patients exhibited significantly reduced hemodynamic parameters compared to group II PH [97].

## 6. Treatment Strategies for PAH and Right-Sided Heart Failure

### 6.1. Current Treatment Options

In patients who do not respond to the initial vasodilator challenge and are resistant to calcium channel blockers such as nifedipine and diltiazem, alternative treatments targeting specific molecular pathways should be considered. Vasoreactivity testing is indicated only in IPAH, HPAH, or drug and toxin-associated PAH, with fewer than 10% of patients in these groups demonstrating a positive response. Patients who initially respond to vasodilator testing require close monitoring to determine if escalation to therapies targeting other pathways is necessary [2,98]. In patients with IPAH, even determined medical treatments often yield unsatisfactory outcomes, leaving double-lung transplantation as the only remaining treatment alternative [99].

Based on the underlying molecular mechanisms, there are four main targets in the treatment of PAH. Stimulation of the NO–soluble guanylate cyclase–cyclic guanosine monophosphate (NO–sGC–cGMP) pathway, activation of prostacyclin (PGI_2_) receptors through agonists or prostacyclin analogs/prostanoids, and antagonism of ET-1 receptors (ETA, ETB, or both), are three different treatment targets and contribute to vasodilatory and anti-proliferative effects in the treatment of PAH [100]. The fourth involves inhibition of activin signaling, which mainly targets cellular proliferation [2].

The effect of phosphodiesterase-5 inhibitors (PDE-5i) on pulmonary vasodilation is well known through their prevention of cGMP degradation. Their use, particularly in PAH associated with connective tissue diseases (such as scleroderma) and IPAH, is well-established [101]. The drugs in this category include sildenafil and tadalafil. The SUPER trial was a randomized clinical trial of PAH patients comparing sildenafil and placebo; the primary endpoint was 6 min walk distance (6MWD). Patients receiving different doses of sildenafil (20 mg, 40 mg, and 80 mg TID) all experienced improved 6MWD. Additionally, the sildenafil group demonstrated an improvement in WHO functional class as well as a reduction in the mPAP. Tadalafil, administered once daily, also improves exercise capacity in PAH patients, along with time to clinical worsening and hemodynamics [102]. Furthermore, based on trials such as the AMBITION study, which assessed upfront combination therapy using tadalafil as the PDE5 inhibitor component, tadalafil is preferred as part of initial combination therapy [98]. Common side effects of both PDE5is include flushing and diarrhea [103].

Another drug within the NO-sGC-cGMP pathway is riociguat, which stimulates the sGC enzyme, augmenting cGMP production. Riociguat is recommended to be used in PAH patients who do not respond well to PDE5 inhibitors. The REPLACE trial demonstrated that PAH patients at intermediate risk of 1-year mortality can achieve clinical improvement when switching from a PDE5i to riociguat [104].

The next group of PH medications is the endothelial receptor antagonists (ERAs). ET-1, a potent vasoconstrictor released from endothelial cells, is increased in patients with PAH. Several clinical trials have evaluated the efficacy of ERAs, including bosentan and macitentan (both dual ETA/ETB receptor blockers), and ambrisentan (a selective ETA receptor blocker) [105]. Compared to placebo, bosentan showed significant improvements in 6MWD and WHO functional class [106,107]. Bosentan has also shown improvement in time to clinical worsening and hemodynamics. Bosentan is used less frequently than macitentan and ambrisentan due to its potential to elevate liver transaminases, necessitating monthly liver function monitoring. It is preferred as a component of initial combination therapy based on evidence from clinical trials. Ambrisentan demonstrated significant improvement in time to clinical worsening as well as the 6MWD [108]. Macitentan is another drug in this category that has been studied in IPAH and CTD-PAH and is effective. In addition, it was used in the PORTICO trial, which was the only RCT in portopulmonary hypertension patients [109]. Among all these drugs, Macitentan and Ambrisentan are preferred over Bosentan [98]. Current meta-analysis revealed that the primary adverse effects associated with ERAs include elevated liver enzymes (notably with bosentan), peripheral edema (observed with both bosentan and ambrisentan), and anemia (linked to bosentan and macitentan) [110].

The third class of medications targets the prostacyclin pathway. PGI_2_ is a vasodilator released by endothelial cells. Synthetic agonists such as epoprostenol, administered intravenously, and treprostinil, available in intravenous, subcutaneous, oral, and inhaled formulations, are commonly used in the treatment of PAH patients. Epoprostenol has demonstrated improvements in exercise capacity and hemodynamic parameters [111], as well as increased survival in a clinical trial involving 81 patients with PAH [112]. The TRIUMPH-1 trial evaluated inhaled treprostinil in PAH patients after 12 weeks of treatment alongside bosentan or sildenafil, showing a significant improvement in the 6MWD [113]. Additionally, the Phase 3b TRANSIT-1 trial found that switching from inhaled treprostinil to oral selexipag was effective, well tolerated by most patients [114]. Common side effects of prostacyclin analogs, regardless of the route of administration, include headache and nausea that have also been observed with selexipag [115].

The fourth and newest treatment includes sotatercept, which is a novel pathway/activin inhibitor. Sotatercept functions as a ligand trap for TGF-β superfamily members, significantly improving the 6MWD and other clinical outcomes [116,117]. The recent Phase III ZENITH trial, presented at the American College of Cardiology, demonstrated that sotatercept significantly reduced all-cause mortality, transplantation rates, and hospitalizations in high-risk PAH patients compared to placebo. The results were so compelling that the trial was stopped early due to demonstrated efficacy (https://www.acc.org/Latest-in-Cardiology/Clinical-Trials/2025/03/28/02/58/zenith) (accessed on 25 May 2025). Several safety concerns were reported with the use of sotatercept compared to placebo. The most commonly reported side effects included epistaxis, telangiectasia, and dizziness. However, the adverse events of interest—predefined parameters used to assess the drug’s safety profile—were primarily bleeding events and thrombocytopenia. Additionally, some patients in the sotatercept group showed increases in hemoglobin levels and red blood cell counts, in contrast to decreases observed in the placebo group [116,117].

Current guidelines favor initial combination therapy, consisting of dual oral treatment with a PDE5i and an ERA for patients who are not classified as high risk. For high-risk patients, initial therapy should include a PDE5i, an ERA, and an intravenous or subcutaneous prostacyclin, based on clinical trial evidence [98]. The AMBITION trial demonstrated that dual therapy with ambrisentan plus tadalafil reduced the composite endpoint of clinical failure compared to monotherapy with either drug [108]. Furthermore, a French registry showed dual therapy with an endothelin receptor antagonist (ERA) and a PDE5 inhibitor was beneficial in terms of WHO functional class and exercise capacity over a 4-month evaluation period [118]. While the TRITON study found no significant difference with initial triple vs. initial dual oral therapy—selexipag (PGI_2_ receptor agonist), macitentan (ERA), tadalafil (PDE5i) vs. placebo with macitentan and tadalafil—it did reaffirm the improvements seen in exercise capacity hemodynamics with initial dual oral therapy with PDE5i and ERA) [119].

**Figure 7:**
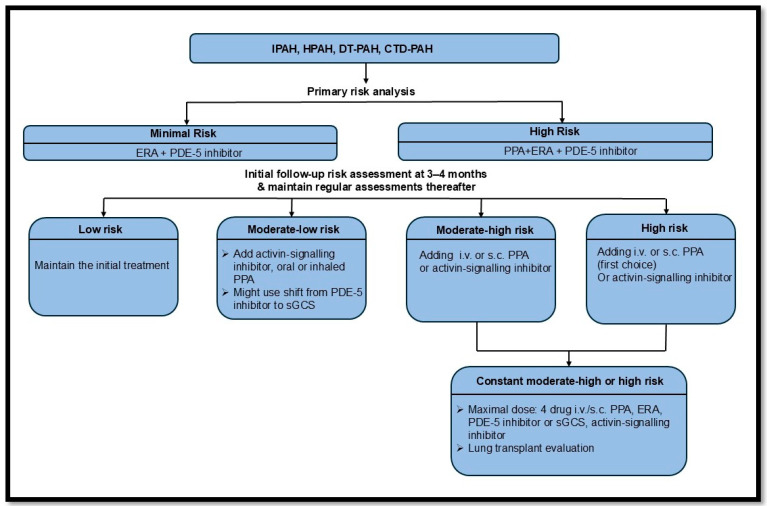
The recent ERS guidelines on pulmonary arterial hypertension treatment [98]. Idiopathic PAH (IPAH); hereditary PAH (HPAH); drug and toxin (DT); connective tissue disease (CTD); endothelin-1 receptor antagonist (ERA); phosphodiesterase-5 inhibitor (PDE-5i); intravenous (i.v.); subcutaneous (s.c.); prostacyclin pathway agent (PPA); soluble guanylyl cyclase stimulator (sGCS).

According to the latest ERS PAH treatment guideline, the optimal PAH therapy involves combination treatment for most PAH groups, typically including ERA and PDE-5 inhibitors for newly diagnosed PAH patients. A detailed algorithm is provided in Figure 7 [98].

### 6.2. Novel/Investigational Therapeutic Approaches

Several new drug classes have been investigated for the treatment of PAH. These focus on reversing vascular remodeling through immune modulation rather than inducing vasodilation [120].

Imatinib and seralutinib are tyrosine kinase inhibitors targeting platelet-derived growth factor receptors (PDGFRs). PDGFR levels are elevated in PAH patients, affecting smooth muscle cell proliferation [121]. The first case report on imatinib in a PAH patient showed significant improvement in that patient, for whom triple therapy had not helped [122]. Subsequently, several clinical trials evaluated the efficacy of imatinib, with the IMPRES trial being the most significant. Patients receiving imatinib demonstrated notable improvements in exercise capacity (6MWT), hemodynamic parameters, and echocardiographic indicators of RV function [123]. However, due to the adverse effects, oral imatinib development was stopped [124], and Phase III trials were recently terminated. The main drawbacks include patient non-compliance and adverse effects, such as subdural hematoma [125]. The IMPAHCT trial evaluated inhaled imatinib (AV-101) in patients with PAH; however, despite good patient compliance, the phase 2b portion of the trial yielded negative results, leading to the discontinuation of the planned phase 3 trial. To date, the full results have not been publicly released (ClinicalTrials.gov; ID NCT05557942).

The phase II study of inhaled seralutinib demonstrated a significant reduction in PVR, which has led to the initiation of an ongoing phase III trial [126]. The phase III study is evaluating the efficacy of seralutinib versus placebo in patients with PAH, with the primary endpoint being improvement in the 6MWD (ClinicalTrials.gov ID NCT05934526).

Recombinant BMP9 therapies, such as MGX292, have shown controversial results in preclinical studies by acting on the native BMPR pathway, and their role in PAH treatment is still not clear [127].

### 6.3. Integrated Imaging and Therapeutic Strategies

CMR findings in PH patients treated with ERAs and PDE5 inhibitors showed significant improvement in RVEF [128]. Also, a reduction in N-terminal pro-B-type natriuretic peptide, which is deemed an alternate indicator of RV wall stress, is significantly lower in the combination therapy of PAH patients [129]. Echocardiographic results confirm that ERAs and PDE5 inhibitors could be beneficial in terms of reducing RV dilation [130].

A key factor that determines prognosis in PAH patients is RV-PA coupling. Some studies have shown the effectiveness of pharmacotherapies on RV pressures [131]. PAH therapy could be beneficial in RV-arterial coupling treatment. ERA, PDE-5 inhibitors, and prostacyclin analogs reduce PVR and RV afterload, improving RV-PA coupling. Such measurements can be obtained from pressure catheterization or noninvasive techniques, particularly CMR or echocardiography, as surrogate approaches [131]. The current method, echocardiography, has shown that the TAPSE/PASP ratio could be a good estimate of RV-PA coupling. Reduced TAPSE (<17 mm) indicates impaired RV systolic function, while increased PASP represents high afterload [132]. A TAPSE/PASP ratio of <0.31 suggests impaired RV contractile function in response to high afterload [54]. Ultimately, RV-specific therapies could be emerging treatments, enhancing RV contractility and adaptation to increased afterload by modulating metabolic pathways and improving energetics [131]. The direct effects of PGI_2_ agonists on the RV and RV-arterial coupling are less investigated. Some studies have shown the beneficial role of these drugs. Inhaled iloprost improves right atrial function, reduces RV dyssynchrony, measured through echocardiography. It has also been observed to reverse RV fibrosis in experimental models of RV failure [133].

Although several studies have been conducted on the efficacy of RV-arterial coupling treatments, most are mainly experimental, and more evidence is warranted to evaluate the reliability of such treatments.

### 6.4. Precision Medicine in PAH Management

Despite all the treatment options available for patients with PAH, the 5-year survival rate remains low. Additionally, PAH is a complex vascular disease with multifactorial etiologies. Current evidence has proven that genetic variations and mutations, as well as differing response rates to existing treatments, underscore the need for precision and individualized treatment options.

Since the role of BMPR2 mutations in IPAH and HPAH is well established, developing risk stratification strategies for patients with these mutations may enable more frequent and effective monitoring. Additionally, family screening and genetic testing allow the identification of at-risk family members, enabling early detection and preventive care. However, genetic testing remains costly and not universally accessible.

As PAH has several subgroups, each group responds differently to current treatments; for example, PAH related to connective tissue disease responds well to anti-inflammatory drugs compared to other forms [134]. Also, some patients with different mutations in the ET-1 gene respond differently to ERA drugs [135].

Currently, two main clinical risk assessment tools are used for PAH: REVEAL 2.0 and COMPERA 2.0. The COMPERA 2.0 model stratifies patients into four risk groups based on three factors, namely, WHO functional class, 6MWD, and serum levels of NT-proBNP, using updated cut-off values [136]. In contrast, the REVEAL 2.0 risk score incorporates a greater number of variables and can predict patient survival more comprehensively [137]. Both tools are available as online applications and are widely used in clinical practice. REVEAL 2.0, in particular, assists clinicians in determining initial treatment strategies and guiding therapy escalation during follow-up.

Phenotyping is another recent approach in terms of PAH that involves characterizing patients based on clinical, hemodynamic, and molecular features. Metabolic phenotypes with abnormalities in glucose and lipid metabolism are emerging as therapeutic targets, such as pioglitazone, which acts as peroxisome proliferator–activated receptor γ (PPARγ) [138]. However, drug-toxin-induced PAH presents unique mechanisms and variable responses to vasodilators such as calcium channel blockers [139]. Thus, comprehensive phenotyping often requires advanced imaging and biomarkers, which may not be widely accessible, and integrating findings into clinical practice remains challenging due to limited trial data.

Furthermore, advances in omics-based precision medicine are enhancing PAH management and include the following: genomics [140]—next-generation sequencing is uncovering novel mutations and pathways; proteomics [141]—biomarkers like NT-proBNP and ET-1 aid in therapy selection and monitoring; and metabolomics [142]—insights into metabolic shifts, such as oxidative stress and mitochondrial dysfunction, are driving the discovery of new drug targets [143].

## 7. Future Directions

Several state-of-the-art imaging modalities have been introduced, such as 4D-flow MRI, which provides better visualization and enhanced diagnostic capabilities. There is a growing trend towards utilizing noninvasive, particularly high-resolution modalities to assess various components of the pulmonary circulation. Photon-counting CT (PCCT), the newest technology introduced, can provide high-resolution images with less radiation dose. Several studies have been conducted on the beneficial use of PCCT [144], particularly for the detection of coronary artery stenosis [145] as well as lung nodules [146]; however, there is no research on its use in PAH patients. PCCT could provide a highly detailed visualization of small pulmonary vessels, aiding in the early detection of distal vascular changes such as wall thickness and lumen narrowing that are often missed with conventional CT. Additionally, PCCT might distinguish between different types of PH, which is a main challenge based on their specific evaluation of perfusion defects or areas of decreased blood flow. Due to the lower radiation dose, PCCT can be utilized for the longitudinal monitoring of PAH patients. Lastly, PCCT can potentially identify new imaging biomarkers for PAH by analyzing tissue composition at a microstructural level, such as changes in calcium or iron.

Additionally, using artificial intelligence, particularly automated analysis of common imaging modalities, including echocardiography or CMR, might detect conventional imaging features of PAH and help clinicians diagnose the condition faster [147]. Lastly, screening patients for common mutations and incorporating genetic and phenotypic screening could enhance the diagnosis of PAH patients.

## 8. Discussion and Conclusions

### 8.1. Summary of Key Findings

Despite advancements in imaging techniques and vascular molecular methodologies, patients with PAH are still often diagnosed late.

The definitive method for diagnosing PAH remains invasive right heart catheterization. Several imaging modalities, such as echocardiography and V/Q scan, have been introduced, which are already part of standard clinical practice. State-of-the-art imaging techniques, particularly MRI with 4D flow sequences, are currently under investigation and may be incorporated into clinical practice in the near future. Also, several clinical trials are underway to evaluate new therapeutic agents, particularly anti-inflammatory drugs. It is most important to find solutions for right-heart failure treatment, in which, in advanced disease, the only option is transplantation.

### 8.2. Importance of an Integrative Approach in PAH Management

It is crucial to incorporate both imaging and molecular diagnostic techniques simultaneously for accurate diagnosis, assessment of disease severity, and evaluation of treatment response. The main prognostic factor for PAH patients is right-sided heart failure. If standard therapies could reverse RV failure, and if imaging modalities—particularly CMR—could be used to re-evaluate treatment response and disease severity, it would greatly benefit patients. Additionally, molecular imaging, as a state-of-the-art technique, might be beneficial in the near future.

## Figures and Tables

**Table 1:** Displays different subgroups of pulmonary arterial hypertension (PAH).

Pulmonary arterial hypertension (PAH):	1. Idiopathic (IPAH): Sparing pulmonary veins	
	2. Heritable: BMPR2 mutations/other mutations	
	3. Drugs\toxins induced: Methamphetamine, Dasatinib (targeted cancer therapy tyrosine kinase inhibitor)	
	4. Associated with other disease (APAH):	4.1. Connective tissue disease Mixed connective tissue disease (MCTD) Systemic lupus erythematosus (SLE)
		4.2. Infection with HIV
		4.3. Portal hypertension Liver disease
		4.4. Congenital heart disease Eisenmenger syndrome, PAH related to systemic-to-pulmonary shunts, PAH associated with small cardiac defects (ASD, VSD), and PAH after cardiac defect closure
		4.5. Schistosomiasis
		4.6. Chronic hemolytic anemia Beta thalassemia Sickle cell disease
	5. Long-term responders to calcium channel blockers (CCBs): Improvement with CCBs	
	6. Features of venous-capillary involvement: Pulmonary veno-occlusive disease	
	7. Persistent PH of the newborn	

**Table 2:** Depicts different imaging modalities in PAH management as well as the advantages and drawbacks of each modality.

Modality		Main Diagnostic Features	Benefits	limitations
Right heart catheterization (RHC)		Direct pressure measurements: mPAP, PVR, PCWP, and cardiac output.	▪ Gold standard method ▪ Comprehensive hemodynamic measurements	▪ Invasive technique ▪ Complications (bleeding, arrhythmia) ▪ Lack of structural and functional information
Echocardiography		▪ RV size and function ▪ PAP via tricuspid regurgitation jet velocity ▪ Septal shift (flattening of interventricular septum) ▪ Pericardial effusion	▪ Available method ▪ Non-invasive ▪ Both functional and hemodynamic information	▪ Operator-dependent ▪ Limited acoustic ▪ Indirect PAP measurement ▪ Limited visualization of pulmonary vessels
V/Q Scan		▪ Rule out other causes of PAH, particularly detection of perfusion defects in CTEPH	▪ High sensitivity for CTEPH ▪ lower radiation exposure ▪ Superior to CT for small perfusion defects	▪ Non-specific findings ▪ Limited anatomical resolution
Computed Tomography (CT)		▪ Dilated PA (higher than 29 mm) or an increased PA-to-aortic ratio (greater than 1). ▪ RV hypertrophy (>4 mm). ▪ RV dilatation (right to left ventricle ratio of >1:1 at mid-ventricular level on axial images).	▪ High spatial resolution ▪ Comprehensive thoracic anatomy ▪ Rapid acquisition ▪ Ruling out CTEPH or interstitial lung disease	▪ Ionizing radiation ▪ Complications: risk of nephropathy ▪ Limited functional information
Cardiac Magnetic Resonance (CMR)	Cine	▪ RV dilation ▪ Increased RV end-diastolic volume (RVEDV) ▪ Reduced RV ejection fraction (RVEF) ▪ Septal flattening or bowing during systole/diastole ▪ Septal flattening (“D-shaped” septum) ▪ Elevated RV/LV volume ratio	▪ Excellent spatial resolution ▪ Comprehensive RV assessment ▪ Non-ionizing exposure ▪ Reproducible ▪ less operator-dependent ▪ Accurate both functional and anatomical assessment	▪ Limited availability ▪ Expensive ▪ Contraindicated in some patients (e.g., pacemakers) ▪ May require contrast (gadolinium) ▪ Requires patient cooperation
	LGE	Fibrosis in RV insertion points or RV free wall.		
	T1 mapping	Elevated T1 relacation time and ECV in diffuse fibrosis		
	Strain/Strain Rate Analysis (Feature Tracking)	Myocardial Deformation, reduced strain rate in three directions, longitudinal, circumferential and radial		
	Flow and Velocity, 4D-flow MRI	Measuring peak and mean velocity in the MPA, LPA and RPA		

Pulmonary arterial hypertension (PAH); Mean pulmonary arterial pressure (mPAP); pulmonary capillary wedge pressure (PCWP); pulmonary vascular resistance (PVR); left ventricle (LV); chronic thromboembolic pulmonary hypertension (CTEPH). Right ventricle (RV); Pulmonary arterial pressure (PAP); Ventilation perfusion (V/Q); Pulmonary artery (PA); Main pulmonary artery (MPA); Left pulmonary artery (LPA); Right pulmonary artery (RPA).
